# Lectures on the Diagnosis and Treatment of Diseases of the Lungs

**Published:** 1841-07-31

**Authors:** W. W. Gerhard


### MEDICAL EXAMINER.

DEVOTED TO MEDICINE, SURGERY, AND THE COLLATERAL SCIENCES.

No. 31.] PHILADELPHIA, SATURDAY, JULY 31, 1841. [Vol. IV.

### LECTURES ON THE DIAGNOSIS AND TREATMENT OF DISEASES OF THE LUNGS.

BY W. W. GERHARD, M. D.

### LECTURE XV.—(*Continued.*)

#### PERICARDITIS.

3. *Auscultation.* The necesssary effect of a large effusion of liquid is to compress the heart and impede the freedom of its action ; the organ is also removed to a greater distance from the walls of the chest, which, of course, makes the sound less distinct to the observer. Hence the natural effect of pericarditis is to render the sounds of the heart distant and feeble ; in some cases to so great an extent that they can scarcely be distinguished. The sounds aie most feeble in those cases in which the effusion is greatest, for if there be lymph and no serum, the diminution of the sounds is much less perceptible, and, in some cases, the first becomes really louder, but more or less altered, and of a bellows or rasping character. The bellows sound is, however, rarely produced by pure pericarditis, but depends, in most cases, upon the inflammation of the internal membrane, though not in all cases, for any great disturbance of the action of the heart may give rise to a bellows sound. The rasping sound is not found in pure pericarditis. The sounds of the heart gradually resume their ordinary distinctness as the inflammation abates, and the effusion is absorbed.

In certain stages of pericarditis another sound occurs, which is similar to that heard in pleurisy under the same circumstances ; it is a slight friction sound, compared by some writers to that produced by the bending of two pieces of new and rather stiff leather. This description is as exact as any other, and if attention be paid to the termination of the systole, and the commencement of the diastole of the heart, it will be readily detected when present. At first it requires some attention to isolate the creaking from the proper cardiac sounds, for it is not very loud in many instances. In other cases it is so distinct as not only to be readily heard, but even to communicate to the hand a decided quivering sensation. The sound may of course occur either in those stages of pericarditis where the liquid is almost absorbed, or in the early stages of the disease, when no serous effusion has taken place.

The local symptoms of pericarditis, other than the physical signs, are extremely irregular ; in many cases the pain, which is often so severe in inflammations of the serous membranes, is totally absent; hence pericarditis has been so frequently overlooked, or the pain may be limited to a very slight feeling of uneasiness, not causing any decided suffering. In a few cases the pain is much more severe, and may become extremely acute, oppressing the action of the heart, and preventing the free motion of the organ; these cases are the severe ones which at one time passed as the type of pericarditis. The dyspnœa is extremely variable, but, as a general rule, is much more moderate in smple cases than in those which are complicated with inflammation of the internal membrane of the heart. As the symptom is not at all limited to pericarditis, it is of little value in diagnosis, and does not often reach an intense degree, except in those cases in which the inflammation is not only extended to both membranes of the heart, but includes the lungs or their membranes.

Cough is a symptom which is rarely absent in the inflammations of the serous membranes of the chest, but it is very slight and short in all of them, and especially in pericarditis, which, of course, involves the lungs only indirectly, and has comparatively little action on the bronchial mucous membrane.

The local signs of pericarditis are, therefore, remarkable for their great irregularity and total want of proportion with the progress of the inflammation or its extent In this respect, pericarditis resembles other serous inflammations, and is even yet more uncertain in its symptoms.

The general symptoms are still more obscure; indeed, no diagnosis can be made from them. In all serous inflammations they are not proportioned to the severity of the local disease; and in pericarditis, the connection of the organ affected with the pulse destroys the value of the symptoms dependant upon it. Obscure and doubtful as it is, the pulse is the only one of the general symptoms which furnishes a guide in pericarditis; the thirst, tne loss of appetite, and cerebral symptoms are completely secondary, and often totally absent; when present, they depend merely on the degree of fever, and are strictly proportioned to it. They may, therefore, be properly passed over without special notice, unless in a monograph which must contain all the symptoms met with in the course of the disease.

The circulation may be perfectly regular, and present nothing anormal both in the arteries and capillary vessels. In a number of cases of pericarditis, especially if of a subacute or scrofulous kind, the pulse does not rise above eighty in the minute, and is soft and regular, while the capillary circulation is equable, and the face scarcely flushed. In other cases of pericarditis, a different effect is produced upon the circulation, the pulse is extremely irregular and very small; these cases are, it is true, generally complicated with endocarditis, but this connexion is not invariable, for pericarditis itself will sometimes so much impede the action of the heart, that the pulse is scarcely felt. Between these opposite conditions of the pulse, there are many intermediate degrees, and often slight irregularity; but, as a general rule, the pulse is smaller than in most other inflammatory diseases, because there is a direct impression made upon the muscular action of the heart. The capillary circulation is at times much loaded, and the face extremely flushed, or even livid, as in other cases in which the circulation through the heart and lungs is much impeded. There is, therefore, nothing peculiar to pericarditis in this condition of things; but, on the contrary, as the variety of symptoms is great, and as the difference in the dyspnœa is such that the disorder varies from a really trifling and latent affection to one attended with the greatest oppression and the most intense anguish, we are obliged, as has been already stated, to look to the physical signs as the only certain means of diagnosis. The other symptoms serve to confirm or to limit the diagnosis, but cannot form the foundation for it.

The prognosis in pericarditis is in general favourable, a very small proportion of cases proving fatal. The exact number cannot be ascertained with accuracy, because so many of the slighter varieties pass unnoticed. Taking the severe and mild forms together, five per cent. would probably be a near approach to an accurate estimate.

The effects of pericarditis are not in general productive of much ultimate mischief, if the disease is not complicated with endocarditis. In this case the real mischief depends upon the latter affection, which leaves behind incurable affections of the valves of the heart. If the pericardium be but slightly inflamed, no possible mischief can result from the slight deposit of lymph or opacity upon its surface; but if the inflammation be extensive enough to leave behind considerable adhesions, they will, to some extent, impede the action of the heart, and thus may favour the formation of organic muscular disease.

The causes of the disorder are either those of ordinary serous inflammations of the chest, or acute articular rheumatism. The latter disease is the more frequent cause of those severe varieties in which there is much pain and other obvious symptoms; but the slighter cases are generally produced by exposure to cold and damp. The renewal of the causes of the disease is apt to produce a repetition of the attacks, until at last the inflammation becomes complicated with endocarditis, or organic alteration of the heart.

The treatment of pericarditis is simple, more so, perhaps, than pleurisy, as the extent of surface involved is less considerable, and the membrane is removed to a very little distance from the surface, which renders the action of local depletion much more certain. The treatment in severe cases which are generally complicated with endocarditis must be extremely active; that is, a large and full blood-letting should be followed by a free cupping or leeching within an hour or two, provided the patient has reacted. If the patient was in previous good health, and the circulation of the skin active, I prefer cups; under other circumstances leeching is necessarily prescribed in preference. The uneasiness is generally relieved, though rarely removed by these first applications. If the pericarditis predominates, it will then be better to continue local bleeding by repeated applications of cups, instead of venesection; but if the inflammation of the internal membrane plays a prominent part, repeated venesection is often necessary in the early periods. In no disease can we trace the powerful and immediate effect of cupping so clearly as in pericarditis; the effusion often diminishes immediately, and the action of the heart becomes fuller and more equable. The greatest benefit does not appear, however, in those cases in which there is much effusion, but rather in the early or dry stages of pericarditis, when there is merely intense vascular excitement, and some secretion of lymph, but very little serum.

If the effusion be large, blisters are a much more effectual means of removing it than cups, or any other vascular depletion. They exert the same power in pericarditis as in other cases of serous effusions, and tend, at the same time, to check the further progress of the inflammation, and favour the absorption of the effused fluid. If the disease becomes chronic, they may be re-applied at short intervals.

The internal medicines are generally less potent than those applied externally. They differ little from those used in other serous inflammations; the most powerful are, of course, the preparations of mercury, pushed so as to produce a rapid ptyalism. In my own practice, I have preferred the combination of calomel, ipecacuanha and opium, to any other form. The ipecacuanha and opium are soothing, and tend to give temporary relief to the patient, while they do not interfere with the action of the mercury, which is the strictly curative agent. The usual dose is a grain of calomel and one of ipecacuanha, with a quarter of a grain of opium, every two hours. If the disease is not violent, the mercury need not be given so frequently. With the commencement of ptyalism, or just before it, there is usually a decided diminution of the symptoms; and often a rapid absorption of the effused liquid takes place.

Digitalis has long been used in the treatment of pericarditis; and with considerable advantage in the chronic forms of the disease, when there is much liquid of a thin serous character. In cases complicated with endocarditis, the digitalis also acts well; but it is so difficult to produce a full and rapid action of the remedy, that it is not safe to trust to it, except as an aid to more efficient means. Tartarized antimony is also of benefit; but as its action is less certain than that of mercury, it should be used only in connection with it, to favour its action, or to diminish the activity of the circulation before the mercury has had time to produce a full impression. Like the digitalis, it is not fitted for those cases in which the internal membrane of the heart is involved.

These measures will generally succeed in violent cases of pericarditis; the slighter ones are relieved by the same treatment, and, indeed, terminate favourably under almost any circumstances.

The hygienic measures necessary in pericarditis are simple enough. Entire rest, abstinence from all stimulants, and almost from food, with a careful avoidance of mental emotions, are necessary in the acute cases. Even in the less violent or more chronic varieties, the patients should carefully avoid such excitants as act especially upon the heart.

The treatment of chronic pericarditis differs but little from that of the acute. General blood-letting is, however, inadmissible, except in a few rare cases, in which the excitement rises nearly to acute inflammation. Repeated cupping, blisters, and occasionally other counter-irritants, are more useful than any other remedies. The repeated, but careful administration of digitalis, is, in this case, of more benefit than when the disease is more acute. Lastly, mercurials in small doses will generally complete the cure.

